# Regulation of Endoplasmic Reticulum Stress-Autophagy: A Potential Therapeutic Target for Ulcerative Colitis

**DOI:** 10.3389/fphar.2021.697360

**Published:** 2021-09-13

**Authors:** Dan Qiao, Ziwei Zhang, Yali Zhang, Qian Chen, Yujun Chen, Yingjue Tang, Xiong Sun, Zhipeng Tang, Yancheng Dai

**Affiliations:** ^1^ Department of Gastroenterology, Shanghai Traditional Chinese Medicine-Integrated Hospital, Shanghai University of Traditional Chinese Medicine, Shanghai, China; ^2^ Institute of Digestive Diseases, LongHua Hospital, Shanghai University of Traditional Chinese Medicine, Shanghai, China; ^3^ Department of Gastroenterology, Shanghai PuTuo District People’s Hospital Affiliated to Tongji University, Tongji University School of Medicine, Shanghai, China

**Keywords:** autophagy, endoplasmic reticulum stress, ulcerative colitis, unfolded protein, unfolded protein response

## Abstract

Ulcerative colitis (UC) is a chronic nonspecific inflammation that mainly affects the mucosa and submucosa of the rectum and colon. Numerous studies have shown that endoplasmic reticulum stress (ERS)-induced autophagy plays a vital role in the pathogenesis of UC. ERS is the imbalance of internal balance caused by misfolded or unfolded proteins accumulated in the endoplasmic reticulum (ER).Excessive ERS triggers the unfolded protein response (UPR), an increase in inositol-requiring enzyme 1, and a Ca^2+^ overload, which activates the autophagy pathway. Autophagy is an evolutionarily conserved method of cellular self-degradation. Dysregulated autophagy causes inflammation, disruption of the intestinal barrier, and imbalance of intestinal homeostasis, therefore increasing the risk of colonic diseases. This review summarizes the pathogenesis of ERS, UPR, and ERS-related autophagy in UC, providing potential new targets and more effective treatment options for UC.

## Introduction

Ulcerative colitis (UC) is a chronic, nonspecific inflammatory disease of the rectum and colon, whose etiology is unexplained. The main symptoms of UC include hematochezia, diarrhea, abdominal pain and tenesmus (Seyedian et al., 2019; Naseer et al., 2020). UC, one of the refractory diseases of the digestive system with recurrent episodes of intestinal inflammation, is common in Western countries, with a prevalence rate of 100∼200/100,000 in Europe and North America. The number of cases reported in China has also increased significantly in recent years (Li et al., 2017; Ng et al., 2018; Feuerstein et al., 2019). UC patients tend to be relatively young, which decreases social productivity and personal quality of life (Kaplan, 2015).

Destruction of the intestinal mucosal barrier caused by the interaction of genetics, infection, immunity, and environmental pollution is the core event leading to the pathogenesis and progression of UC. Accordingly, as a major component of the intestinal mucosal barrier, damage to intestinal epithelial cells (IECs) may play a decisive role in this event (Ren et al., 2019; Yan et al., 2020). IECs, including microfold (M) cells, enteroendocrine cells, absorptive epithelial cells, goblet cells, and Paneth cells, respond to various types of immune cells, and regulate epithelial barrier function and gut microbiota (Okumura and Takeda, 2017; Soderholm and Pedicord, 2019). IECs, with a well-developed structure of the endoplasmic reticulum (ER), are one of the most metabolically exubera-nt cell types. Sustained and severe endoplasmic reticulum stress (ERS) induces autophagy through the unfolded protein reaction (UPR) in IECs, which causes inflammation. Excessive ERS can also disrupt the intestinal mucosal barrier, and ultimately lead to UC (Hosomi et al., 2015; Iida et al., 2017; Ma et al., 2017). This review is a systematic appraisal of the current literature to provide a better understanding of the role of the pathogenesis of ERS, UPR, and ERS-related autophagy in UC.

## ERS in UC

The ER is one of the largest cellular organelles, and has a complex structure (Lu et al., 2020). It is the main site of protein synthesis, folding, lipid synthesis, carbohydrate metabolism, and calcium storage (Schwarz and Blower, 2016; Stevenson et al., 2016). ERS is driven by the accumulation of unfolded and misfolded proteins in the ER (Liu and Green, 2019).

During ERS, the cell activates a response to changes in protein folding, which is called the UPR (Hetz, 2012). Moreover, the other pathway is ER-associated degradation (ERAD), which maintains ER (Olzmann et al., 2013). However, persistent ERS and UPR can induce cell death (Bernales et al., 2006). To date, the UPR is initiated by three kinds of ER transmembrane sensors, including inositol-requiring enzyme 1 (IRE1), protein kinase R-like ER kinase (PERK), and activating transcription factor 6 (ATF6) (Ron and Walter, 2007). The interaction between the heavy-chain-binding protein (BiP) and adenosine nucleotides mainly participates in these three processes (Pobre et al., 2019). Under ERS conditions, BiP dissociation activates IRE1, PERK, or ATF6, and initiates cascades of the UPR and downstream signals (Ma et al., 2017). Above all, three UPR signaling pathways (IRE1, PERK, and ATF6) are involved in the pathogenesis of UC.

## IRE1 Pathway

IRE1 is a key factor in the severity and duration of UPR (Pincus et al., 2010). Mammalian IRE1 contains two subtypes: IRE1α and IRE1β. IRE1α is widely expressed in the human body, while IRE1β is mainly expressed in the gastrointestinal tract and pulmonary epithelial cells (Tirasophon et al., 1998; Wang et al., 1998). X-Box-binding protein 1 (XBP1) in mammals is a crucial transcriptional activator in this process. The increase in protein load in the ER activates XBP1 (Calfon et al., 2002), which can relieve ERS. Simultaneously, IRE1 can also bind to and activate tumor necrosis factor receptor–associated factor 2 (TRAF2), which is a binding protein that binds plasma membrane receptors to c-Jun N-terminal kinase (JNK), and then activates JNK (Urano et al., 2000). Regulated IRE1-dependent decay (RIDD) has dual functions: maintaining homeostasis under low ERS and inducing apoptosis by excessive ERS (Pluquet et al., 2013).

IRE1β is expressed in goblet cells, which secrete mucoprotein 2 (MUC2). MUC2 is more easily degraded by pathogens in the colon environment during UC, suggesting that MUC2 acts as a protective mucin in UC. The level of MUC2 increases sharply in IRE1β^−/−^ mice, indicating that IRE1β can degrade MUC2 and maintain the stability of MUC2 in the intestine (Tsuru et al., 2013). Bertolotti found that IRE1β^−/-^ mice developed colitis several days earlier than wild-type mice with dextran sulfate sodium (DSS) induced UC, indicating that IRE1β^−/−^ mice had a marked susceptibility to DSS (Chassaing et al., 2014). IRE1α gene deletion induced the apoptosis of IECs, which destroyed the intestinal mucosal barrier and led to spontaneous colitis (Zhang et al., 2015). Therefore, IRE1 is an essential signal in the pathogenesis of UC, and its absence leads to spontaneous colitis.

MicroRNAs are also involved in UPR/ERS through IREIα, an ER transmembrane kinase-endoribonuclease (RNase). Activation of IRE1α caused decay of select microRNAs (miRs -17, -34a, -96, -125b), which inhibit translation of Caspase-2 mRNA generally. IRE1α regulated Caspase-2 translation via downregulating select anti- Caspase-2 miRNAs by cleaving select pre-miRNAs to prevent proper DICER processing of their mature forms. Thus, IRE1α cleaves select microRNAs to prevent the translation of proapoptotic Caspase-2 during ERS (Upton et al., 2012). Besides that, IRE1α induces thioredoxin-interacting protein (TXNIP) to activate the NLRP3 inflammasome and promote cell death during ERS. However, TXNIP mRNA stability during ER stress is controlled by a specific micro-RNA, miR-17. miR-17 levels decline speedily under ERS. IRE1α increases TXNIP mRNA stability by reducing miR-17. And TXNIP protein activates the NLRP3, causing Caspase-1 cleavage and interleukin 1β (IL-1β) secretion. Therefore, microRNAs indirectly regulates signaling hubs to control cell death during ERS (Lerner et al., 2012).

Transcription of the XBP1u gene regulates the ERS-mediated UPR signaling pathway. Studies found that ERS increased and goblet cells decreased in Xbp1^−/−^ mice, which decreased MUC 2 secretion and enhanced susceptibility to experimental colitis. The expression of tumor necrosis factor (TNF-a) and C/EBP homologous protein (CHOP) increased, while the antimicrobial ability decreased (Kaser et al., 2008). Briefly, IRE1β, IRE1α, and XBP1 in the IRE1 pathway are associated with a protective effect on UC by degrading MUC2 secreted by goblet cells, protecting the intestinal mucosal barrier, improving the sensitivity of mice to DSS, maintaining the homeostasis of the intestinal environment and inhibiting the inflammation process.

## PERK Pathway

During ERS, PERK oligomerization and autophosphorylation activate eukaryotic initiation factor 2α (eIF2α) kinase and alleviate ERS by augmenting the UPR (Ma et al., 2002; Verfaillie et al., 2012). Moreover, the expression of ATF4 was induced by phosphorylation of eIF2α (Vattem and Wek, 2004). Subsequently, the expression of CHOP increased and induced apoptosis. (Rozpedek et al., 2016). X-linked inhibitor of apoptosis protein (XIAP) is a potent inhibitor of cysteinyl aspartate specific proteinase (Caspase) activity (Eckelman et al., 2006). ATF4 promotes the degradation of XIAP, while the PERK signaling pathway downregulates XIAP synthesis through two modes: 1) reduction of the synthesis of XIAP through the phosphorylation of eIF2α and 2) degradation of XIAP through ATF4 activation (Hiramatsu et al., 2014). In addition, ATF4 induces the expression of ATF5, which can promote apoptosis (Teske et al., 2013). The repression of PERK signaling blocks the expression of most genes by overcoming ERS, and leads to cell death (Mcquiston and Diehl, 2017).

The extension of eIF2α phosphorylation results in an increase in ATF4 and CHOP expression. Moreover, high expression of CHOP mediates apoptosis in epithelial cells, which promotes the progression of UC (Waldschmitt et al., 2014). Furthermore, CHOP also stimulates MAC-1 to promote macrophage infiltration and induce reactive oxygen species (ROS) production in macrophages by upregulating endoplasmic reticulum oxidoreductin 1α (ERO-1α) (Namba et al., 2009). eIF2α phosphorylation activates nuclear factor kappa-B (NF-κB) signaling, therefore activating more inflammatory factors and disrupting the intestinal mucosal barrier (Deng et al., 2004). Okazaki found that inhibiting the dephosphorylation of eIF2α could inhibit the PERK signaling pathway and alleviate DSS-induced colitis (Okazaki et al., 2014). Specifically, the PERK pathway induced the expression of CHOP and ATF5, and degraded XIAP through ATF4. This mechanism promoted apoptosis and raised proinflammatory cytokines, which disrupted intestinal epithelial function and affected the development of UC.

## ATF6 Pathway

The ATF6 transcription factor contains two subtypes: ATF6α and ATF6β. In the process of ERS, ATF6, released from the ER membrane, is cleaved by proteases in the Golgi apparatus and transferred to the nucleus. In the nucleus, ATF6 can bind specific DNA and initiate a series of signals to maintain ER homeostasis (Wang et al., 2000; Sato et al., 2011). In addition, ATF6 also regulates the expression of CHOP, which in turn regulates ERS (Yang et al., 2020). ATF6α induces the phosphorylation of Akt and activates the NF-κB pathway (Yamazaki et al., 2009). The absence of the SIP ATF6-processing enzyme and mutation of the S1P-encoding gene (Mbtps1) resulted in increased susceptibility to DSS-induced colitis (Brandl et al., 2009). The inhibition of ATF6α signaling can significantly generate the expression of IL-8 and TNF-α proinflammatory cytokines (Stengel et al., 2020). Therefore, the ATF6 pathway can enhance the expression of inflammatory cytokines and aggravate intestinal inflammation by activating the NF-κB signaling pathway and by expressing genetic mutations, which exacerbate the development of UC.

## ERS, Immune Response and UC

ERS is responsible for the development of UC through a variety of immune responses. Related studies have found that IL-22, dendritic cells (DCs), and nucleotide-binding oligomerization domain (NOD) exhibit potential effects on the intestinal immune response. IL-22, combined with IL-17A, regulate transcription during ERS and promote apoptosis in IECs. The IL-22-ERS axis is vital in the pathogenesis of chronic colitis and might provide a new therapeutic target for future treatment (Powell et al., 2020). Interestingly, William found that ERS stimulated cloned human colorectal gland cells to produce more IL-8, and activated dendritic cells to become proinflammatory cells. This indicated that there was a previously unknown mechanism between epithelial ERS and immune activation in inflammatory bowel disease (IBD) (Rees et al., 2020). In addition, NOD-like receptors (NLRs) were pattern recognition receptors (Caruso et al., 2014). Marijke found that after thapsigarnin treatment, the NOD1/2 level and IL-6 production increased sharply. IL-6 production was significantly decreased in bone marrow–derived macrophages (BMDGs) of dithiothreitol-induced NOD1/2^−/−^ mice compared with wild-type mice. In the NLR family, NOD1 and NOD2 induce ERS to produce more IL-6 through the IRE1α/tumor necrosis factor receptor–associated factor 2 (TRAF2) pathway, causing intestinal inflammation (Keestra-Gounder et al., 2016). Recent studies on ERS, the immune response and UC have shown that ERS can induce apoptosis in IECs and promote the expression of proinflammatory cytokines by regulating the expression of IL-22, DCs, and NOD during the immune response, thereby causing intestinal inflammation and accelerating the development of UC.

**FIGURE 1:**
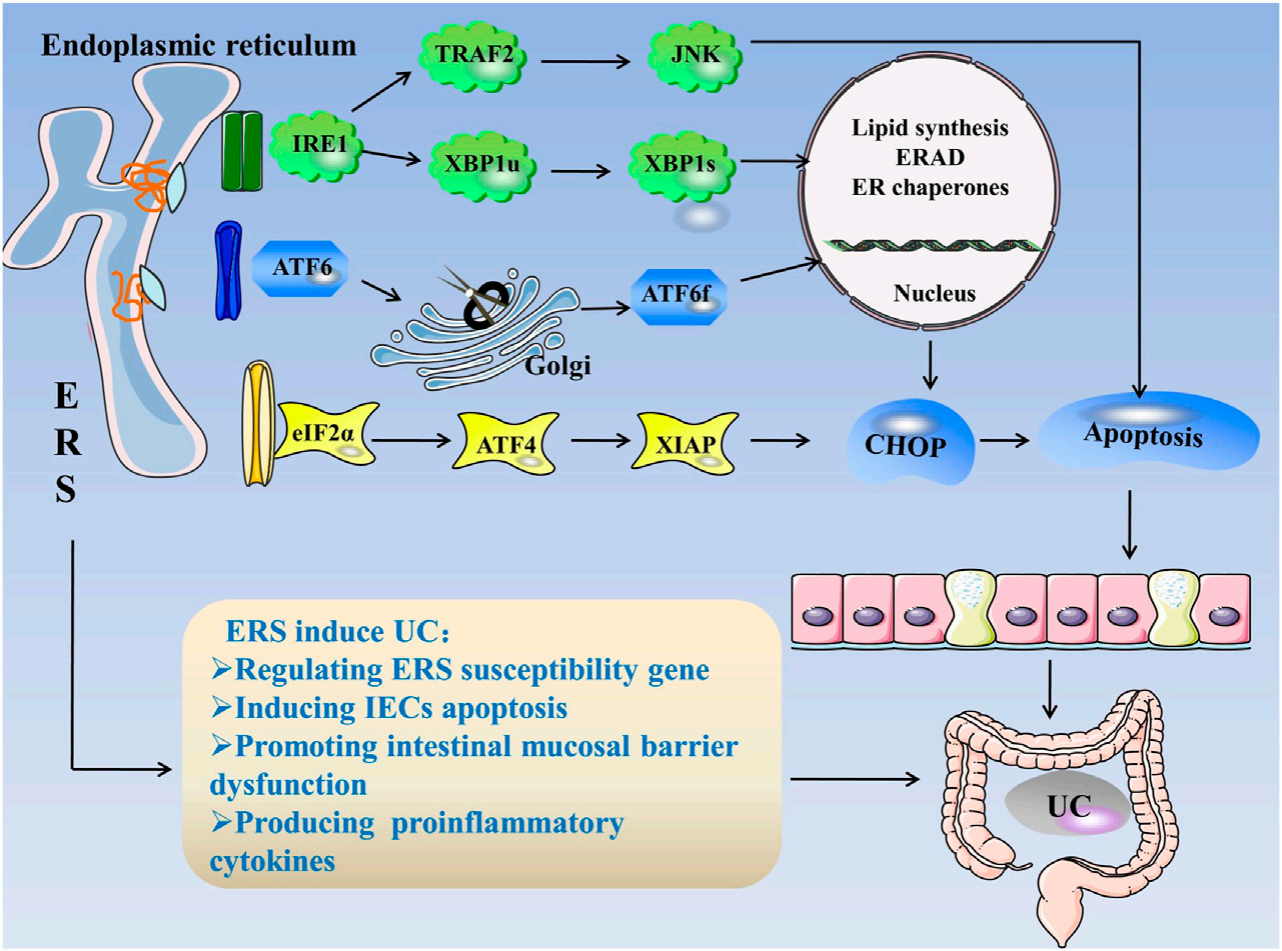
ERS in UC. When unfolded or misfolded proteins accumulate excessively in the ER, ERS occurs and the UPR is initiated. The UPR consists of three pathways: IRE1, PERK, and ATF6. When the cell is in steady state, the three stress-related proteins bind to GRP78. When ERS occurs, GRP78 dissociates from three kinds of receptors and activates the IRE1, PERK, and ATF6 pathways. 1) IRE1 cleaves XBP1u into more stable XBP1s, and IRE1 can bind to TRAF2 to activate JNK. The factors related to ER folding, lipid biosynthesis, and ERAD are regulated during the IRE1 reaction. 2) PERK activates eIF2α through autophosphorylation, eIF2α activates ATF4, and ATF4 induces CHOP expression, which is a cytokine that promotes apoptosis. 3) ATF6 is cleaved in the Golgi apparatus and binds to specific DNA to regulate CHOP. ERS regulates ERS susceptibility genes, induces IEC apoptosis, destroys the intestinal mucosal barrier, and produces proinflammatory cytokines, leading to UC. **Abbreviations:** ATF, activating transcription factor; CHOP, C/EBP homologous protein; ER, endoplasmic reticulum; ERS, endoplasmic reticulum stress; ERAD, endoplasmic reticulum associated degradation; eIF2α, eukaryotic initiation factor 2α; GRP78, glucose-regulated Protein 78; IECs, intestinal epithelial cells; IRE1α, inositol-requiring enzyme 1; JNK, c-Jun N-terminal kinase; PERK, protein kinase R-like endoplasmic reticulum kinase; TRAF2, tumor necrosis factor receptor–associated factor 2; XBP1, X-box-binding protein 1; UC, ulcerative colitis; UPR, unfolded protein response.

Consequently, the mechanism of ERS causing UC can be summarized as follows: 1) regulation of ERS susceptibility genes, 2) induction of apoptosis in IECs, 3) intestinal mucosal barrier dysfunction, and 4) production of pro-inflammatory cytokines, which induces intestinal inflammation and the occurrence of UC (Figure 1).

## Autophagy in UC

Autophagy is an evolutionarily conserved process, whose main function is to degrade endogenous biological macromolecules for recycling (Ravanan et al., 2017). In the case of nutritional deficiency, autophagy is rapidly induced by self-digestion to maintain cell vitality, and core anabolic functions are promoted under conditions of adequate nutrition (Kaur and Debnath, 2015; Yang et al., 2019). Three kinds of autophagy are found in mammals: macroautophagy, microautophagy and chaperone-mediated autophagy (Dikic and Elazar, 2018). Recent studies have reported that autophagy is regulated by autophagy-related genes (ATG) (Li and Zhang, 2019), mammalian target of rapamycin (mTOR) (Munson and Ganley, 2015), adenosine 5′-monophosphate-activated protein kinase (AMPK) (Li and Chen, 2019), Ca^2+^ (Hu et al., 2019) and NOD2 (Negroni et al., 2016).

ATG is involved in the formation of multiple autophagosomes, starting with the activation of the unc-51 like autophagy activating kinase 1 (ULK1) complex (Kabeya et al., 2000). Similar to the mammalian homolog of Atg8, microtubule-associated protein light chain 3 (LC3) has two forms: LC3I and LC3-II. The level of LC3-II reflects the number of autophagosomes. Recently, LC3 is a classic indicator of autophagy in mammals (Tanida et al., 2008). Paiva demonstrated that the level of LC3II is higher in UC. A decreased number of cells exhibiting colocalized LC3/p62, which was verified by immunofluorescence, indicates that autophagy is involved in the pathogenesis of UC (Paiva et al., 2018). In DSS-induced colitis, the number of Lgr5+ stem cells, the LC3II/I ratio and the level of p62 increase, which is aggravated by activating autophagy in Lgr5^+^ stem cells (Xie et al., 2020). Ardali found that the level of ATG5 closely relates to autophagy in the stool of UC patients and is significantly higher than that in healthy people (Ardali et al., 2020). This finding indicates that autophagy plays a central role in the pathogenesis of UC and might be used as a diagnostic marker for UC in the future.

After activation, the ULK1 mammalian autophagy complex binds to vesicles and phosphorylates ATG9. Next, at the amplification stage, ATG8 (LC3) combines with phosphatidylethanolamine (PE) to form an ATG8–PE complex, which promotes the elongation of the autophagic membrane, and the closure and formation of autophagosomes and autophagosome lysosomes (Lin and Hurley, 2016; Goodwin et al., 2017; Yu et al., 2017; Levine and Kroemer, 2019; Turco et al., 2020). ATG9A decreases in DSS-induced colitis, and the overexpression of ATG9A improves autophagy induced by rapamycin (Xu et al., 2018). The aforementioned studies indicate that ATG9 and ATG5 autophagy-related genes might be important genes in the pathogenesis of UC, thus assisting in clinical diagnosis.

mTOR forms the catalytic subunits of two different protein complexes, mTOR complex 1 (mTORC1) and 2 (mTORC2), which play an important role in protein synthesis, lipid and glucose metabolism and other physiological functions (Saxton and Sabatini, 2017). The initiation of autophagy requires the Beclin1–VPS34 core complex, and mTORC1 negatively regulates autophagy by inhibiting the ULK1 and VSP34 complex (Rabanal-Ruiz et al., 2017). Death-associated protein 1 (DAP1) has been identified as a novel substrate of mTOR. DAP1 negatively regulates autophagy by inducing apoptosis and reducing the number of autophagosomes with PERK-eIF2α (Yahiro et al., 2014). mTOR can also regulate autophagy and further regulate UC in various ways. In DSS-induced colitis, deficiency of meteorin-like protein (METRNL), secreted by IECs, deteriorated UC partially by inhibiting autophagy through the AMPK-mTOR-p70S6K pathway. METRNL deficiency aggravated UC by inhibiting autophagy, suggesting that UC can be attenuated by activation of autophagy (Zhang et al., 2020). Hypoxia inactivates mTOR and degrades p62 and LC3, reducing inflammation by restoring autophagy (Cosin-Roger et al., 2017). Zhou found that boosting mTOR-dependent autophagy through the NF-κB pathway quenches intestinal inflammation. The mTOR inhibitor AZD8055 (ATP-competitive mTOR inhibitor) alleviates experimental colitis in mice (Zhou et al., 2018). Therefore, the inhibition of mTOR may help alleviate the symptoms of UC by activating autophagy.

MicroRNAs (miRNAs) are noncoding RNAs that are indirectly involved in autophagy and through inhibition of Beclin1 (Chen et al., 2017). Schaefer found that the expression of miRNAs in the blood and tissues of the UC group and the expression of miR-19a, miR-21, miR-31, and miR-101 were significantly increased (Schaefer et al., 2015). This indicates that miRNAs are involved in the pathogenesis of UC and that specific miRNAs distinguish UC from other diseases. Wang found that miRNAs regulate NF-κB or mTOR signaling to modulate autophagy in intestinal cells by releasing anti- or proinflammatory factors (Wang et al., 2018). Based on these studies, miRNAs regulate autophagy in a variety of ways, including disruption of the intestinal mucosa and changes in intestinal permeability to aggravate or improve UC, but the specific role of miRNAs still needs to be verified.

Vitamin D receptor (VDR) is closely related to autophagy. VDR plays a vital role in IECs by reducing apoptosis and enhancing autophagy through Beclin-1 (Lu et al., 2019). Zhang found that VDR deficiency promoted the release of the tight junction protein Claudin-2, which enhanced the permeability of the intestinal mucosal barrier and further accelerated the progression of UC (Zhang et al., 2018). Yongyan Shi found that necroptotic apoptosis was one of the pathogeneses of IBD. VDR inhibits necroptotic apoptosis, alleviates inflammation, and suppresses the induction of colitis by preventing receptor interacting serine/threonine kinase 3 (RIPK3) from binding to RIPK1 (Shi et al., 2020). Jot and others showed that VDR also protects the intestines through the VDR-gut microbiota axis and reduces the susceptibility of DSS-induced colitis (Ooi et al., 2013). These studies all indicate that VDR enhances autophagy and alleviates colitis through different signaling mechanisms.

**FIGURE 2:**
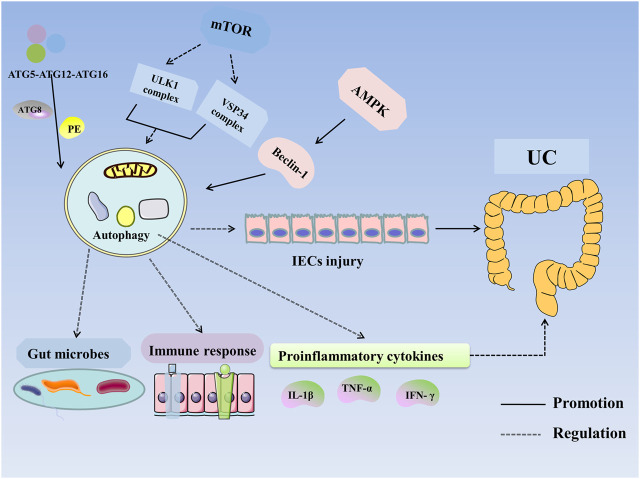
Autophagy in UC. Autophagy is regulated by ATG, mTOR, AMPK, and NOD2. ATG participates in the formation of the ULK1 complex and initiates autophagy. The ATG12–ATG5–ATG16 complex recruits autophagosome membranes and combines ATG8 (LC3) with phosphatidylethanolamine (PE) to form an ATG8–PE complex, leading to the formation of autophagosomes and autophagolysosomes. mTOR negatively regulates autophagy by inhibiting the ULK1 complex, VSP34 complex, and DAP1. AMPK activates ULK1 and phosphorylates Beclin-1 to activate autophagy. VDR enhances autophagy through Beclin-1. Autophagy improves UC by altering the expression of susceptibility genes, improving intestinal microbes and the immune response and inhibiting the expression of proinflammatory cytokines. **Abbreviations:** AMPK, 5′-monophosphate-activated protein kinase; ATG, autophagy associated gene; IECs, intestinal epithelial cells; IFN-γ, Interferon-γ; IL-1β, interleukin-1β; mTOR, mammalian target of rapamycin; PE, phosphatidylethanolamine; TNF-α, tumor necrosis factor-α; UC, Ulcerative colitis.

In summary, autophagy can restore the intestinal mucosal barrier and alleviate UC by regulating expression of susceptibility genes, modifying intestinal microbes, inhibiting proinflammatory cytokines, and suppressing the immune response (Figure 2).

## ERS and Autophagy

Under oxidative stress, energy deficiency, Ca^2+^ depletion, increased mRNA translation, metabolic changes and inflammatory stimulation, cell homeostasis is destroyed and ERS is activated (Luo and Cao, 2015). Several mediators released under ERS can directly induce the formation of autophagosomes and initiate autophagy (Kaser and Blumberg, 2011). Autophagy plays two major functions: on the one hand, moderate autophagy maintains the stability and survival of cells; on the other hand, excessive autophagy causes cell damage and even apoptosis (Kaur and Debnath, 2015). ERS induces the transformation of LC3 from LC3-I to LC3-II and the formation of autophagy (Ogata et al., 2006; Wu et al., 2021).

Recent studies have confirmed the connection between ERS and autophagy, and ERS induces autophagy through various pathways. For example, PERK regulates ATF4 and CHOP transcription factors, influences autophagosome formation, and further affects autophagy (Rouschop et al., 2010). B cells controlled by Trk-fused gene (TFG) are more sensitive to ERS and contain more LC3 and an increased number and size of autophagosomes (Steinmetz et al., 2020). ERS upregulates death-related protein kinase 1 (DAPK1) through ATF6, mediates mAtg9 trafficking, and activates autophagy (Zhou et al., 2016). In addition, ATF4 induces the expression of DNA damage response 1 (REDD1) during ERS. The upregulated expression of REDD1 in UC is correlated with autophagy induction by inhibiting mTOR (Kimball and Jefferson, 2012; Angelidou et al., 2018).

ERS can induce autophagy not only through the UPR pathway, but also by Akt signal transduction. For instance, ROS mediate Akt inactivation and increase the expression of ERS-related molecules such as CHOP and XBP1, leading to apoptosis. ERS can induce autophagy and reduce inflammation by inhibiting the phosphatidylinositol (3-kinase PI3K)/serine-threonine protein kinase (Akt)/mammalian target of rapamycin (mTOR) pathway (Xue et al., 2017; Chung et al., 2019). This indicates that ERS and autophagy can be activated by inhibiting the Akt pathway. ERS induced an increase in CHOP expression and a decrease in B-cell lymphoma-2 (Bcl-2) expression, and activated autophagy by releasing Beclin-1 through the PERK/CHOP/Bcl-2/Beclin-1 pathway (Liu et al., 2014; Ning et al., 2019). Corazzari found that ERS activates the Tribbles homolog 3 (TRB3) axis and the IRE1/TRAF2/apoptosis signal regulating kinase-1 (ASK1)/JNK signaling axis to induce autophagy (Corazzari et al., 2015). IRE1/XBP1 and IRE1/JNK1 both induce autophagy by activating Beclin-1 (Rather et al., 2020).

In general, ERS induces autophagy through multiple pathways: 1) the IRE1/TRAF2/ASK1/JNK/Bcl-2 (Vps34) signaling axis; 2) the IRE1/XBP1/Beclin-1 signaling axis; 3) the Akt/CHOP/Beclin-1 signaling axis; 4) the ATF4/REDD1/mTOR signaling axis; and 5) the PERK/CHOP/Bcl-2/Beclin-1 signaling axis. Whether ERS-induced autophagy plays a role in more signaling axes, for example, ATF6–XBP1–ULK1 and ATF4–ATG7–Beclin-1, needs to be further explored.

## ERS, Autophagy and UC

The interaction between ERS and autophagy can synergistically affect the development of UC. Additionally, IECs, as a target of ERS and autophagy, play a vital role in the pathogenesis of UC (Adolph et al., 2013).

On the one hand, ERS regulates autophagy. Recent reports indicate that the mechanism by which ERSregulates autophagy is complex. Lopes found that ERS activated the ATF6–DAPK1 signal in IECs and enhanced autophagic killing of bacteria (Lopes et al., 2018). The expression of REDD1 in intestinal neutrophils is closely related to the severity of UC. REDD1 activates neutrophil autophagy by inhibiting mTOR phosphorylation, and autophagy positively regulates neutrophil extracellular traps (NETs). Intestinal neutrophil expression through NETs promotes the release of IL-1β, which mediates inflammation and further exacerbates UC. The REDD1/autophagy/NETs/IL-1β pathway plays an important role in the initiation and propagation of UC. Therapy targeting IL-1β could be beneficial for active UC (Angelidou et al., 2018). The expression of glucose-regulated protein (GRP)78 in the colon is increased in UC, especially in the inflamed intestinal mucosa (Shkoda et al., 2007). Tréton found that unspliced and spliced XBP1, GRP78 and GRP94 and ER degradation in the colonic mucosa of UC patients is associated with elevated levels of the active form of ATF6 (p50ATF6α) and impairment of the eIF2α–ATF4–CHOP pathway (Tréton et al., 2011). Stengel found that ATF6α, as an intermediate signaling molecule that regulates its upstream regulators and downstream XBP1 and Atg16L1, participates in the crosstalk between ERS and autophagy of IECs in IBD (Stengel et al., 2020). ERS activates autophagy through multiple signals in IECs and aggravates UC.

On the other hand, autophagy regulates ERS. Moderate autophagy can inhibit ERS, increase the number of goblet cells and mucin secretion, protect intestinal epithelial mucosal barrier function, and relieve the symptoms of UC. In DSS-induced colitis, the inhibition of triggering receptor expressed on myeloid cells 1 (TREM1) induced macroautophagy and chaperone-mediated autophagy, which compensates for the UPR to reduce ERS (Kökten et al., 2018). In addition, the stimulation of NOD and TNF receptors in IECs promote ATG16L1 stabilization via conserved helix-loop-helix ubiquitous kinase (IKKα)-dependent phosphorylation of ATG16L1 at serine 218, thereby protecting ATG16L1 against caspase-3-dependent degradation and limiting ERS activation (Diamanti et al., 2017). In fact, compared with Atg16L1^[ΔIEC]^ or Xbp1^[ΔIEC]^ mice, mice lacking Atg16L1 and Xbp1 (Atg16l1^[ΔIEC]^Xbp1^[ΔIEC]^) develop more severe colitis. However, all Atg16L1^[ΔIEC]^, Xbp1^[ΔIEC]^ and Atg16L1^[ΔIEC]^Xbp1^[ΔIEC]^ mice show increased accumulation and excessive activation of ER to nucleus signaling 1 (ERN1), due to the deficiency of ERN1 degradation caused by autophagy (Tschurtschenthaler et al., 2017).

**FIGURE 3:**
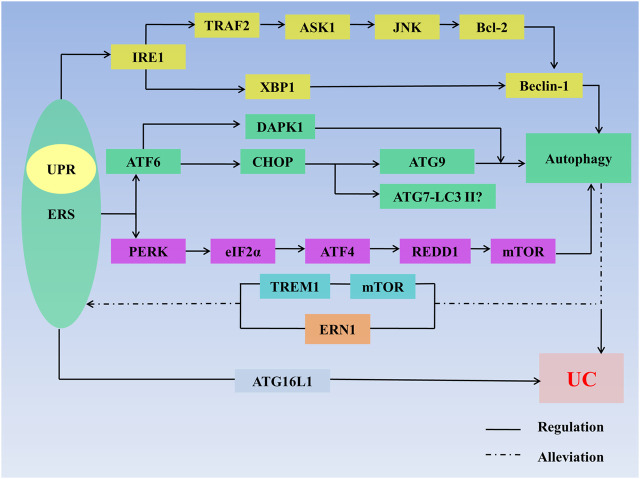
ERS, autophagy, and UC. ERS regulates autophagy through multiple pathways: 1) IRE1/TRAF2/ASK1/JNK/Bcl-2/Beclin-1 signaling axis; 2) lIRE1/XBP1/Beclin-1 signaling axis; 3) ATF6/CHOP/ATG9 signaling axis; 4) ATF6/DAPK1 signaling axis; 5) ATF6/DAPK1 signaling axis; 6)ATF6/CHOP/ATG7-LC3II signaling axis; 7) ATF6/CHOP/ATG9 signaling axis; and 8) PERK/elf2α/ATF4/REDD1/mTOR signaling axis. ERS regulates UC through defects in the ATG16L1 gene. Autophagy stimulates mTOR receptors in IECs to inhibit TREM1 and through regulating ERN1 to alleviate ERS. Autophagy also regulates UC. The interaction between ERS and autophagy synergistically affects the development of UC. **Abbreviations:** AMPK, 5′-monophosphate-activated protein kinase; ASK 1, apoptosis signal regulating kinase-1; ATF, activating transcription factor; ATG, autophagy associated gene; Bcl-2, B-cell lymphoma-2; CHOP, C/EBP homologous protein; DAPK1, death-related protein kinase 1; eIF2α, eukaryotic initiation factor 2α; ER, endoplasmic reticulum; ERAD, endoplasmic reticulum-associated degradation; ERN1, endoplasmic reticulum to nucleus signaling 1; ERS, endoplasmic reticulum stress; LC3Ⅱ, microtubleassociated protein light chain 3II; mTOR, mammalian target of rapamycin; IRE1, inositol-requiring enzyme1; JNK, c-Jun N-terminal kinase; PERK, protein kinase R-like endoplasmic reticulum kinase; TRAF2, tumor necrosis factor receptor–associated factor 2; TREM1, triggering receptor expressed on myeloid cells 1; XBP1, X-Box-binding protein 1; UC, ulcerative colitis; UPR, unfolded protein response.

In general, moderate ERS can maintain intestinal homeostasis, but when ERS is too strong, key molecules such as IRE1, PERK and ATF6 which produce proinflammatory cytokines and destroy the intestinal mucosal barrier to induce UC, are lacking. Autophagy can inhibit the expression of proinflammatory cytokines and improve the immune response and can also reduce UC in other ways, but excessive autophagy increases cell apoptosis and aggravates UC (Figure 3).

The inhibition of autophagy can be used as a therapeutic strategy in UC. Many drugs can inhibit autophagy. The mTOR inhibitor 2-chloro-N-(6-cyanopy-ridin-3-yl) propanamide alleviated UC symptoms in mice by inhibiting autophagy (Bhonde et al., 2008). Curcumin reduced the number of autophagosomes by regulating autophagy and improved the symptoms of DSS-induced colitis in mice (Yue et al., 2019). Nicotine increased the expression of LC3II/LC3I and Beclin-1 through the AMPK–mTOR–p70S6K pathway, reduced p62 levels, and relieved experimental colitis by regulating autophagy (Gao et al., 2020). Oral administration of Na_2_SO_3_ and NaHSO_3_ (3:1) produces SO_2_.SO_2_ suppresses autophagy by reducing oxidative stress and reducing the expression of the proinflammatory cytokines TNF-α, IL-1β, and IL-6, as well as downregulating Beclin1 expression. Therefore SO_2_ alleviates the pathological manifestations of colitis in rats by anti-inflammatory, antioxidation and autophagy inhibition (Banerjee et al., 2019). Galangin pretreatment increases autophagy-related protein expression and promotes the formation of autophagosomes, which can be used to prevent UC (Xuan et al., 2020). Melatonin alleviates colitis-related colon cancer by reducing autophagy, as revealed by the expression of autophagy-related markers (Trivedi et al., 2016). These drugs all reduce UC inflammation by regulating the expression of autophagy-related proteins.

## ERS, Autophagy Related Drugs for UC

Autophagy deficiency is closely related to the pathogenesis of UC. Some therapeutic drugs work by improving autophagy, which makes autophagy a new therapeutic target for the treatment of UC. Drugs that modulate both ERS and autophagy need further investigation (Hooper et al., 2016). Berberine (BBR) inhibits the IRE1/XBP1 pathway and JNK activation and reduces the expression of proinflammatory cytokines and ERS. Hence, BBR might be one of the targeted therapeutic agents for UC (Hao et al., 2012). *Lactobacillus paracasei*–derived extracellular vesicles (LpEVs) attenuate DSS-induced colitis by reducing the expression of pro-inflammatory cytokines IL-1α, IL-1β, IL-2, and TNF-α proinflammatory cytokines and promoting the expression of anti-inflammatory cytokines, including IL-10 and TGFβ. LpEVs increase IRE1 and PERK phosphorylation, ATF6 cleavage, and CHOP expression by activating ERS to reduce LPS-induced intestinal inflammation and maintain intestinal homeostasis (Choi et al., 2020). Low-dose naltrexone reduces the level of ERS, restores the intestinal mucosal barrier, and improves the severity of IBD (Lie et al., 2018). Lycium barbarum leaves contain a variety of compounds and prevent inflammation through the IRE1–XBP1–dependent ER stress pathway, which might be linked to its antioxidant activity, making the leaves a beneficial food for human health (Lee et al., 2021). Dodecapeptide (LR12) is an inhibitor of triggering receptor expressed on myeloid cells-1 (TREM-1). Compared with healthy mice, the mice in the DSS^[+]^/LR12^[+]^ group were similar and had no signs of ulcerations. However, DSS^[+]^/LR12^[−]^mice developed ulcers with inflammatory cell infiltration, and increased endoscopic scores. LR12 reduced ERS and restored impaired autophagy, indicating that TREM-1 inhibition alleviates the symptoms of colitis, mitigates endoscopic tissue damage, and prevents UC (Kökten et al., 2018). Hooper found that azathioprine induces autophagy by regulating mTORC1 signal transduction and the UPR sensor PERK, thus contributing to its efficacy in treating IBD (Hooper et al., 2019). The aforementioned drugs can reduce or prevent UC by inhibiting ERS.

## Conclusion and Prospects

The role of ERS and autophagy in IBD has received increasing attention. This study summarizes the relationship between ERS and autophagy in UC. Moderate ERS can maintain the balance of the intestinal environment, but excessive ERS can induce intestinal inflammation by regulating ERS susceptibility genes, inducing intestinal epithelial cell apoptosis and intestinal mucosal barrier dysfunction, and producing proinflammatory cytokines, leading to the occurrence of UC. However, ERS can activate autophagy, which alleviates UC by inhibiting proinflammatory cytokines and immune responses and improving the intestinal microorganisms, but excessive autophagy still aggravates UC. The interaction between ERS and autophagy can synergistically affect the development of UC. Exploring the important mechanism of ERS-induced autophagy in the pathogenesis of UC can contribute to the understanding of the pathogenesis of UC and provide an effective method for the treatment of UC in the future. However, the specific regulatory mechanism of the ERS–autophagy signaling axis needs further investigation. For example, it is of interest to determine whether there are more stress- and autophagy-related pathways; the specific role and exact function of each protein, molecule, and enzyme in the respective pathways and to identify more autophagy-related genes and their regulatory mechanism in UC. The activation of key molecules in ERS can activate autophagy through multiple pathways. However, the following issues remain unaddressed: whether inhibition of one of the key molecules can affect multiple pathways and will produce side effects; if the effect of single target treatment is not significant, whether multitarget treatment can be used; how each signaling pathway works in multitarget therapy; whether the therapeutic drugs confirmed in current studies that can regulate ERS-autophagy in mice can play the same role in other organisms; and whether the combination of targeted autophagy and targeted ERS agents can exert better effects. There are many drugs for treating UC, but some patients have not been cured. Hence, it is urgent to develop novel drugs for the treatment of UC. A better understanding of the relationship among ERS-induced autophagy in UC, regulation of ERS-autophagy, alleviation of IEC damage, restoration of intestinal mucosal barrier function, and maintenance of intestinal homeostasis can provide potential new targets and more effective therapy for UC.
